# Supplementary material for: Effective Treatment of Patients Experiencing Primary, Acute HIV Infection Decreases Exhausted/Activated CD4+ T Cells and CD8+ T Memory Stem Cells
Source: Cells. 2022 Jul 27;11(15):2307. doi: 10.3390/cells11152307 (PMC9367582; doi:10.3390/cells11152307)
Supplement: Supplementary file 1 [file cells-11-02307-s001.zip › Figures S1-S7.pdf]

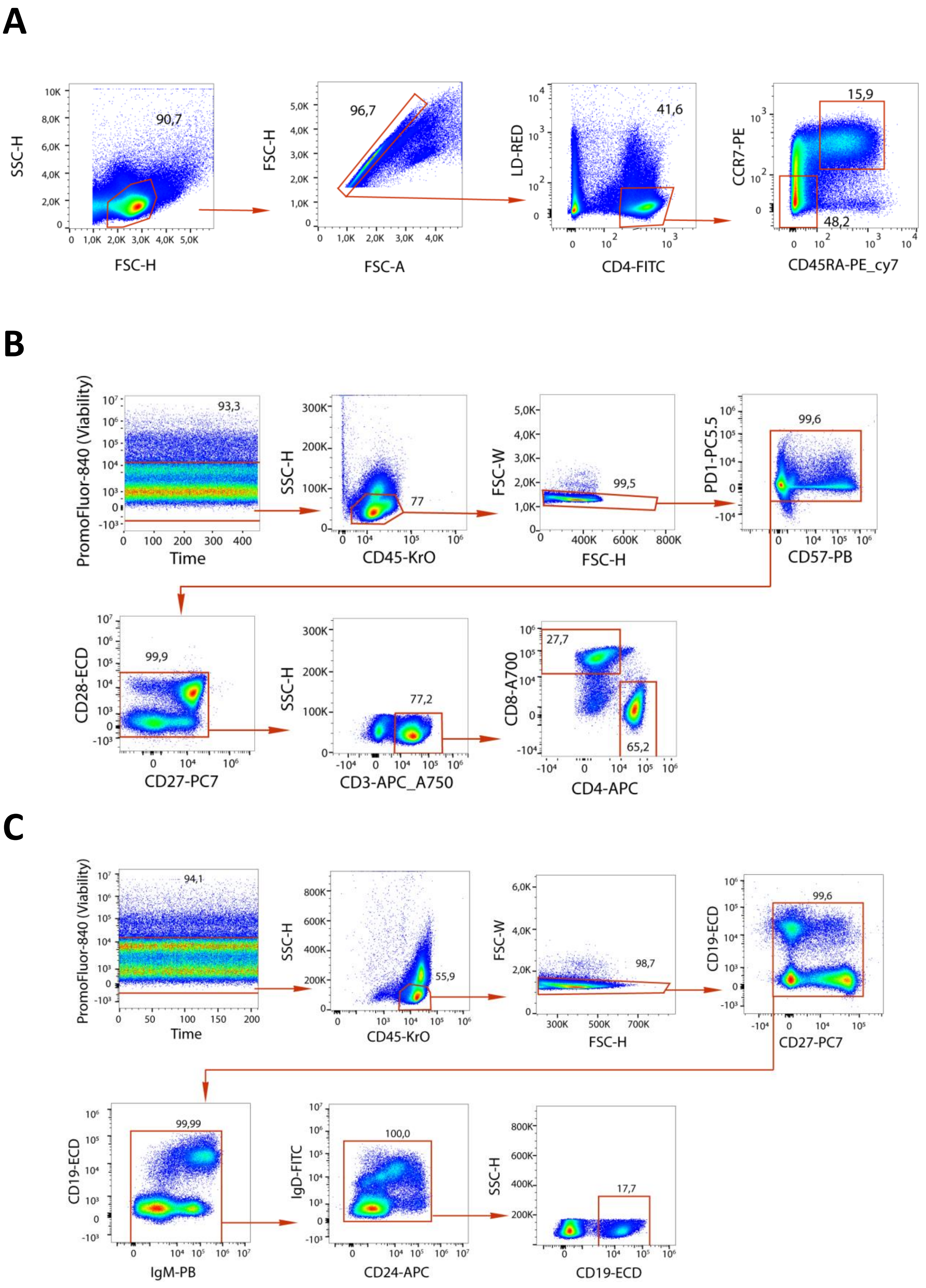

Figure S1. Representative gating strategies

(A) Gating strategy used to identify and isolate viable naïve (CD45RA+CCR7+) and effector memory (CD45RA−CCR7−) within CD4+ T cells. (B) Representative gating strategy used to identify CD4+ and CD8+ T cell among PBMC. (C) Representative gating strategy used to identify B cells among PBMC. Selected CD4+, CD8+, CD19+ main populations were analyzed by unsupervised clustering analyses.

A

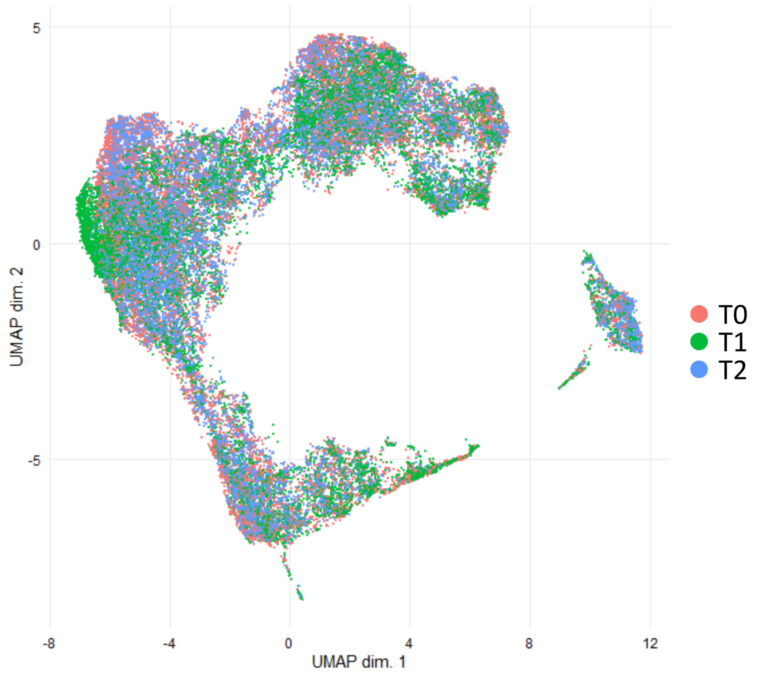

B

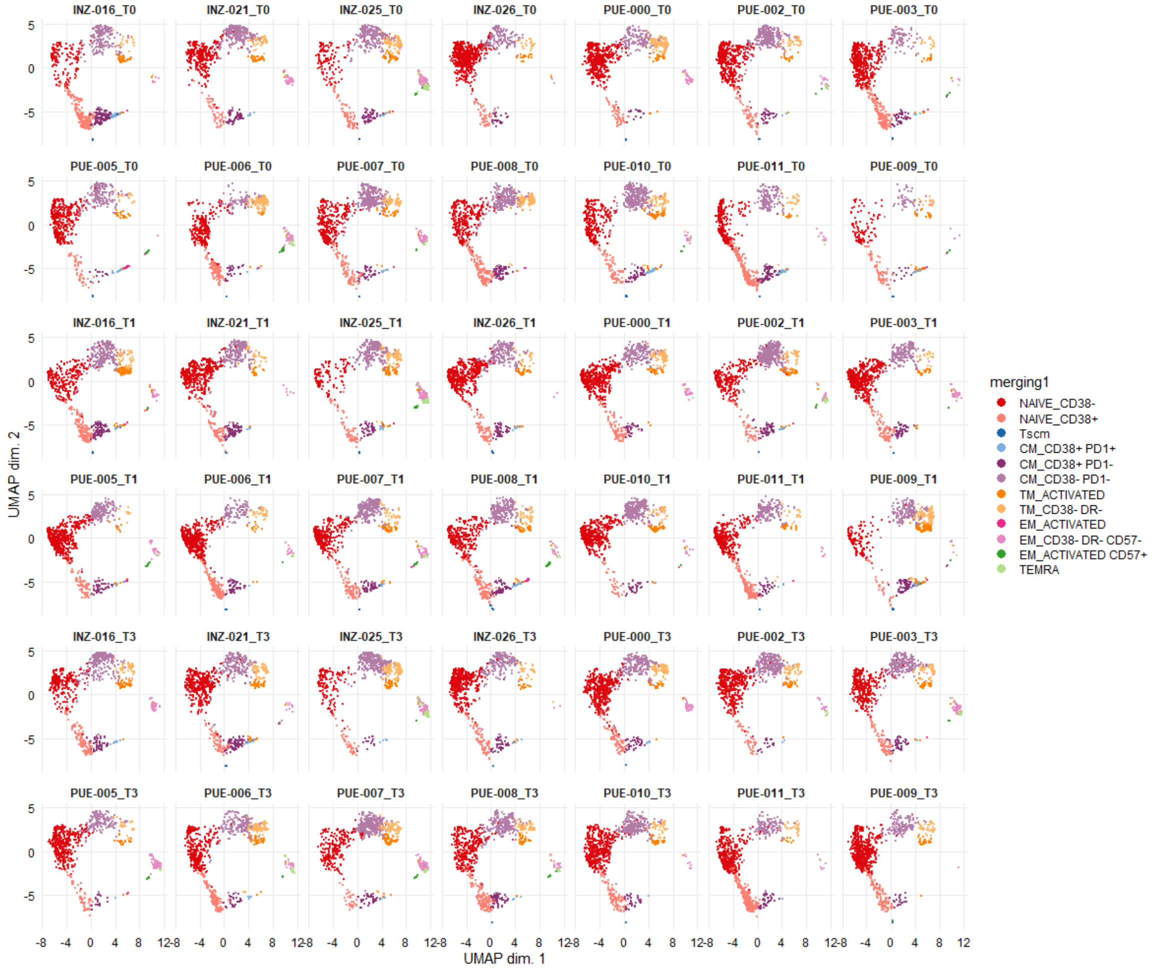

Figure S2. Quality control CD4+ T cells

(A) UMAP graphs of CD4+ T cells overlaid for multiple condition; (B) Projection of UMAP graph stratified by sample.

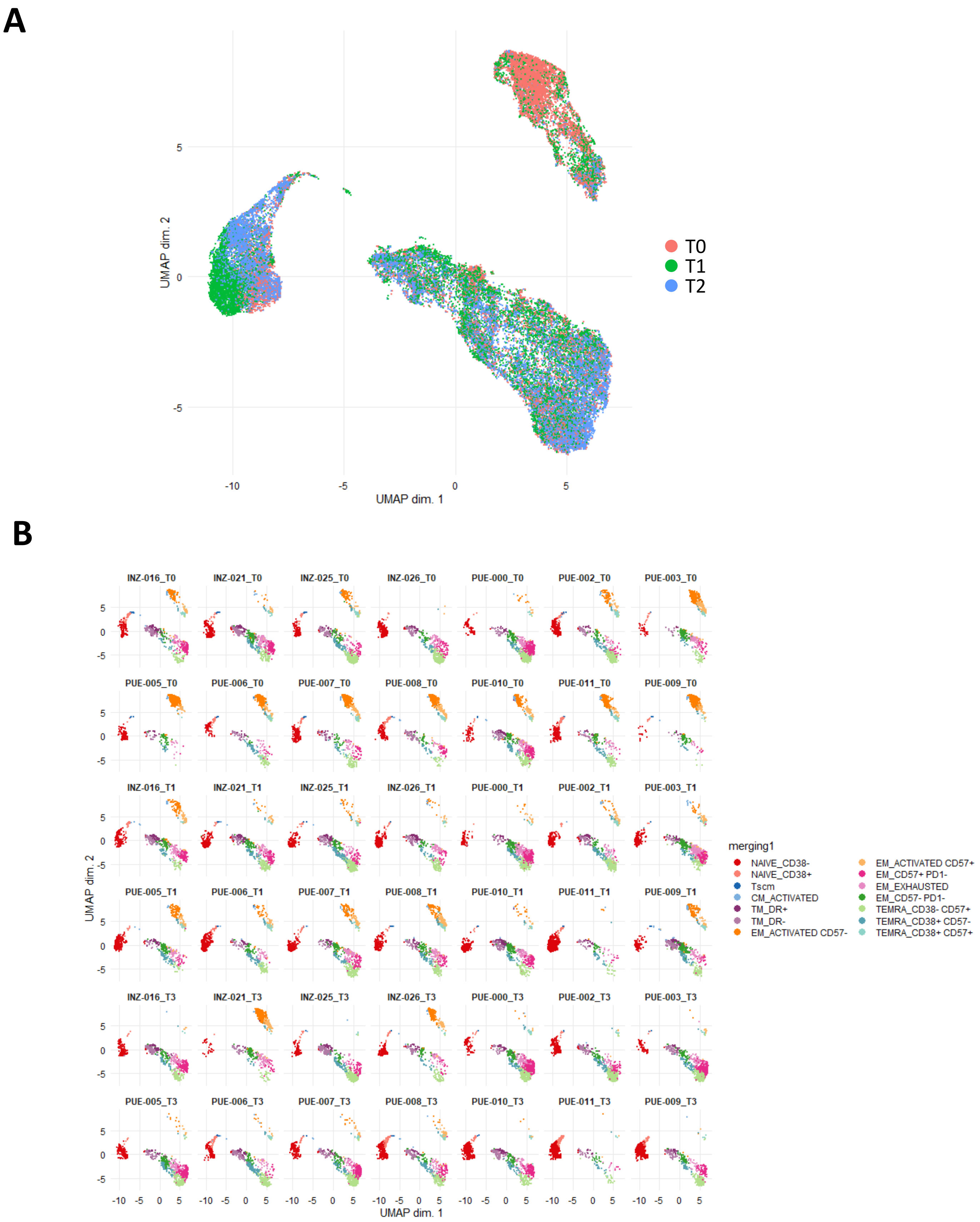

Figure S3. Quality control CD8+ T cells

(A) UMAP graphs of CD8+ T cells overlaid for multiple condition; (B) Projection of UMAP graph stratified by sample.

A

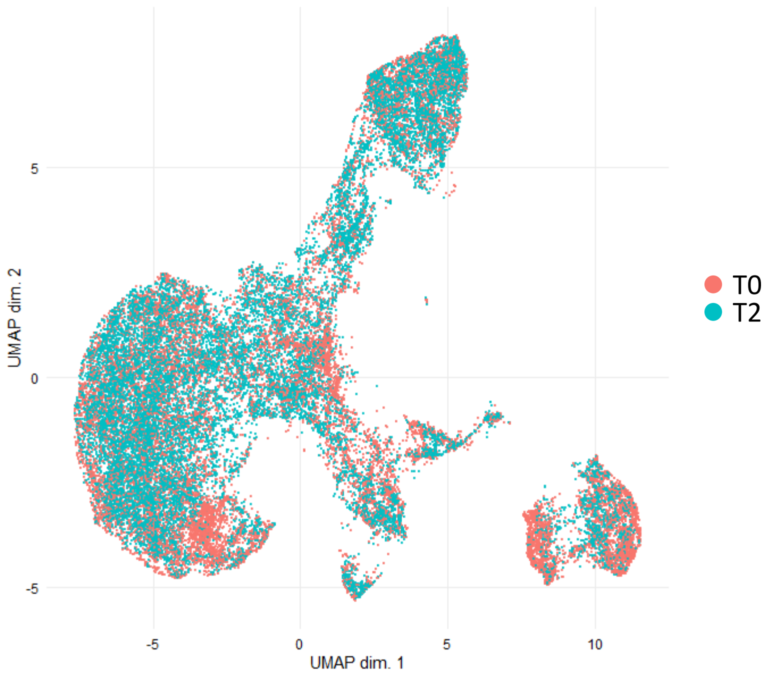

B

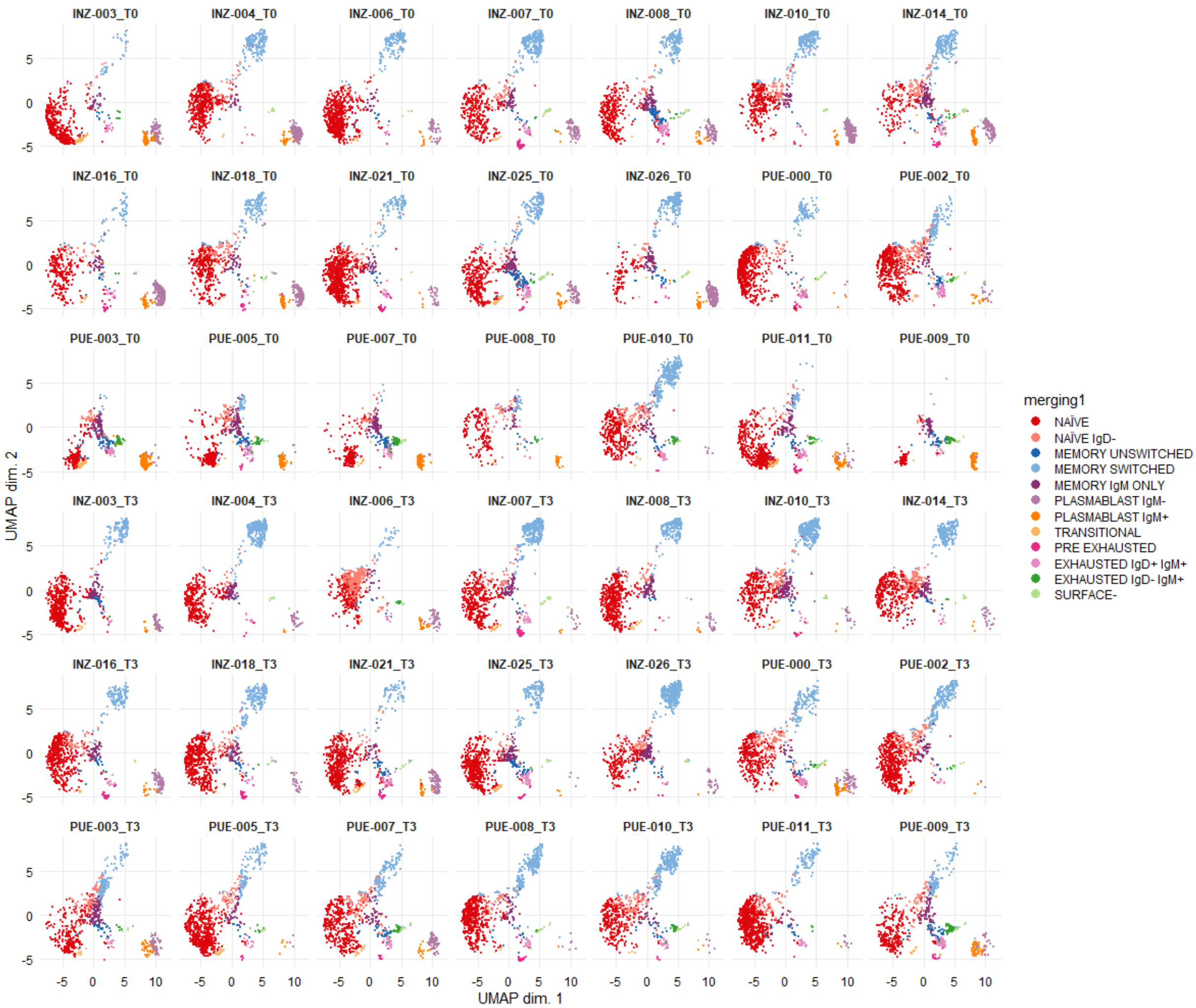

Figure S4. Quality control CD19+ B cells

(A) UMAP graphs of CD19+ B cells overlaid for multiple condition; (B) Projection of UMAP graph stratified by sample.

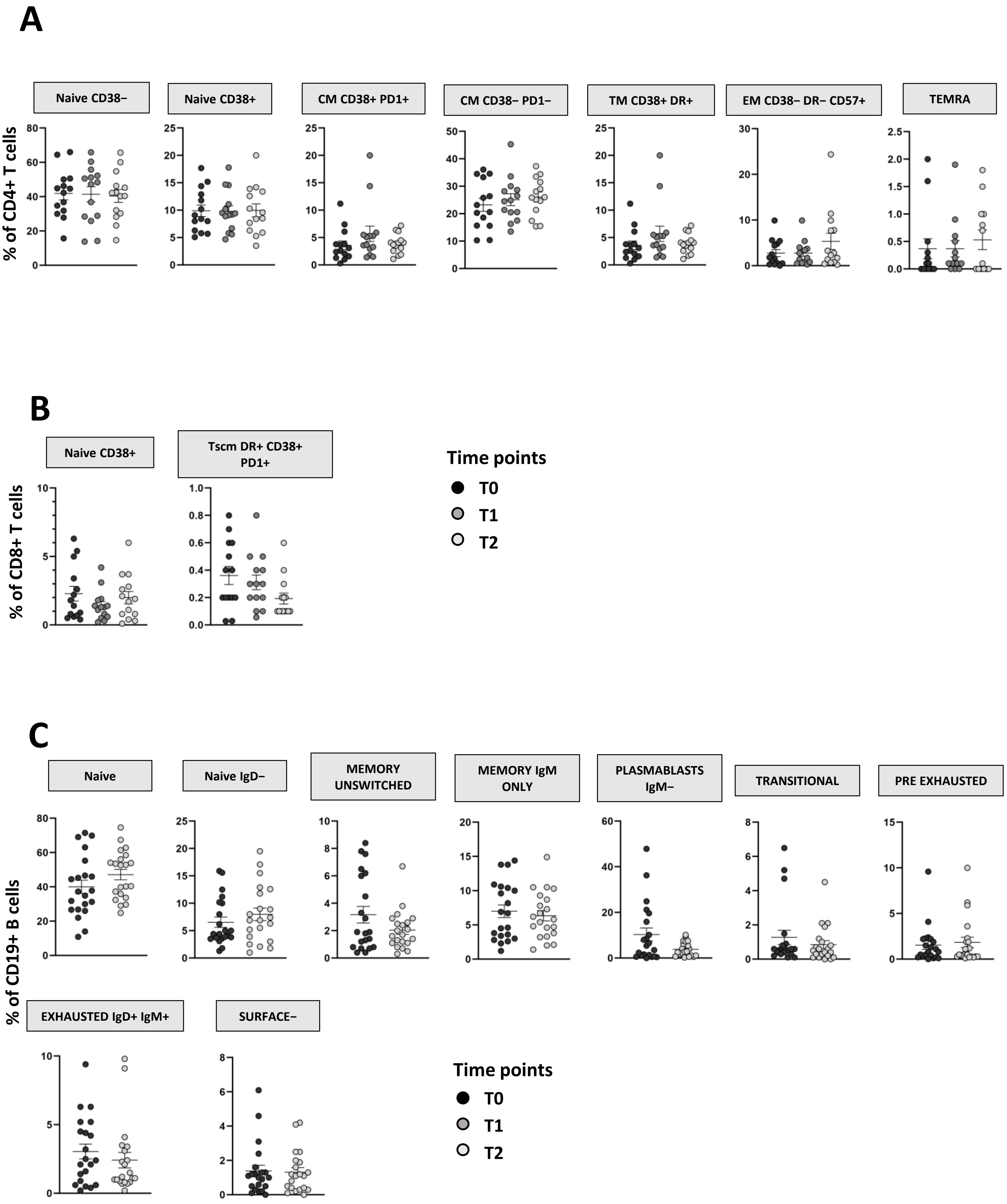

Figure S5. Dotplots showing the percentage of cells whose percentage remains unchanged after therapy initiation. (A) CD4+ T cells; (B) CD8+ T cells and (C) CD19+ B cells. T0 (black circles; n=14), T1 (grey circles; n=14) and T2 (lightgrey circles; n=14) timepoints. The central bar represents the mean ± SEM. The adjusted p-values were obtained by patient pairing generalized linear mixed model (GLMM) that compares cluster percentages at T0, T1 and T2 (threshold: FDR≤0.05).

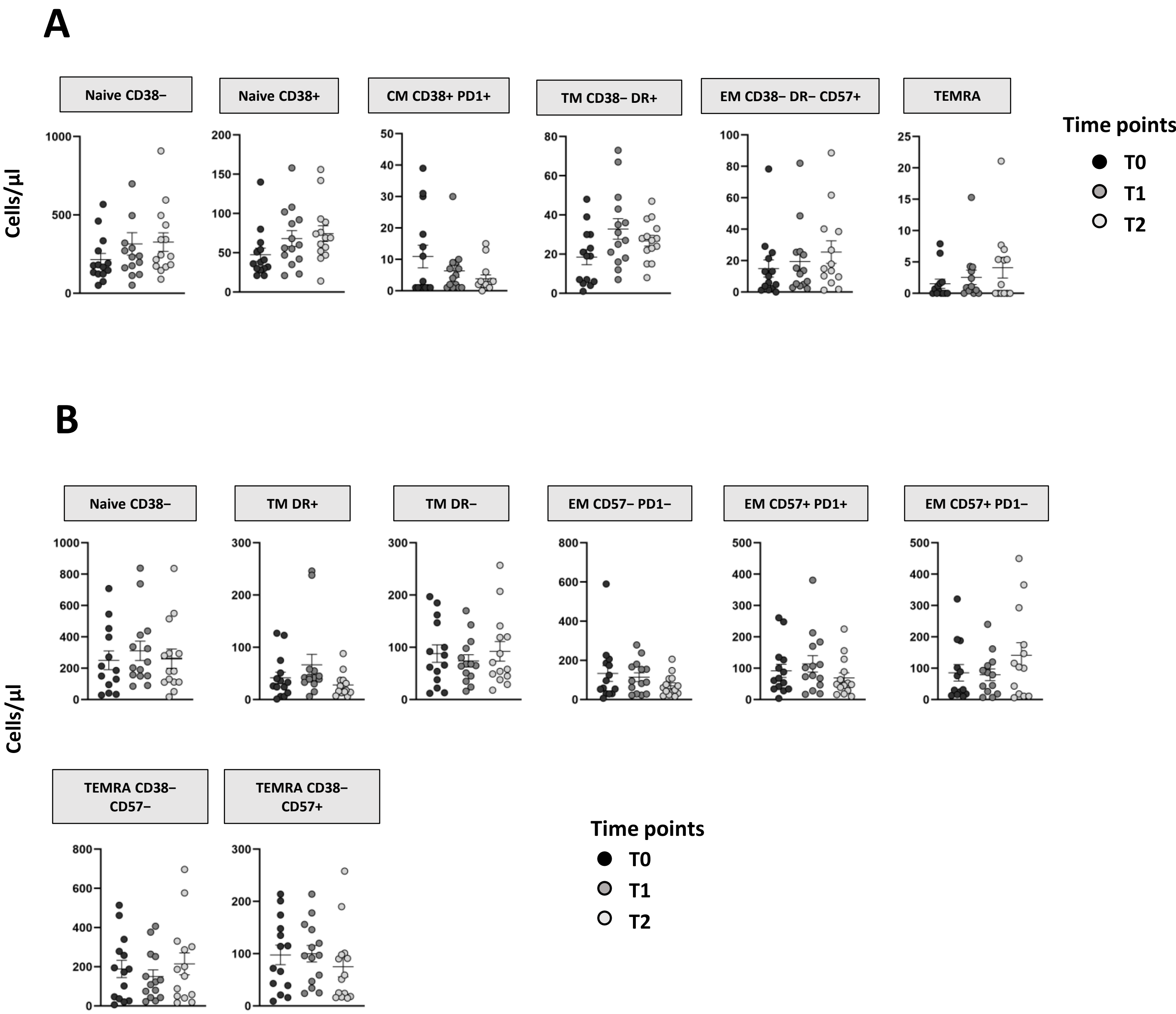

Figure S6. Dotplots showing the absolute number of cells whose remains unchanged after therapy initiation. (A) CD4+ T cells; (B) CD8+ T cells. T0 (black circles; n=14), T1 (grey circles; n=14) and T2 (lightgrey circles; n=14) timepoints. The central bar represents the mean ± SEM. The adjusted p-values were obtained by patient pairing generalized linear mixed model (GLMM) that compares cluster percentages or absolute count at T0, T1 and T2 (threshold: FDR≤0.05).

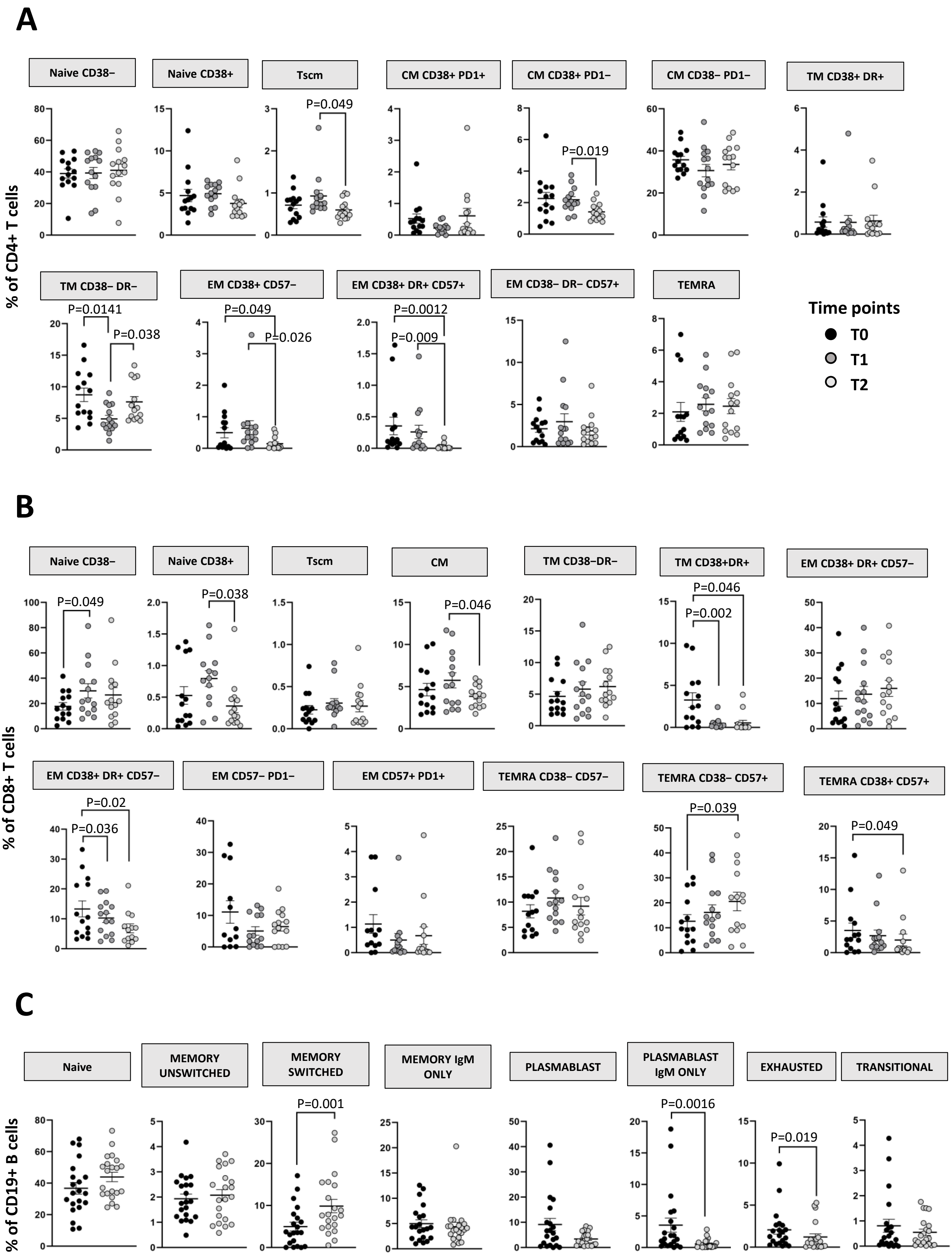
A
% of CD4+ T cells
Naive CD38−
Naive CD38+
Tscm
P=0.049
CM CD38+ PD1+
CM CD38+ PD1−
P=0.019
CM CD38− PD1−
TM CD38+ DR+
TM CD38− DR−
P=0.0141
P=0.038
EM CD38+ CD57−
P=0.049
P=0.026
EM CD38+ DR+ CD57+
P=0.0012
P=0.009
EM CD38− DR− CD57+
TEMRA
Time points
T0
T1
T2
B
% of CD8+ T cells
Naive CD38−
P=0.049
Naive CD38+
P=0.038
Tscm
CM
P=0.046
TM CD38−DR−
TM CD38+DR+
P=0.046
P=0.002
EM CD38+ DR+ CD57−
EM CD38+ DR+ CD57−
P=0.02
P=0.036
EM CD57− PD1−
EM CD57+ PD1+
TEMRA CD38− CD57−
TEMRA CD38− CD57+
P=0.039
TEMRA CD38+ CD57+
P=0.049
C
% of CD19+ B cells
Naive
MEMORY UNSWITCHED
MEMORY SWITCHED
P=0.001
MEMORY IgM ONLY
PLASMABLAST
PLASMABLAST IgM ONLY
P=0.0016
EXHAUSTED
P=0.019
TRANSITIONAL

Figure S7.

Dotplots showing the relative cells percentage obtained by regular manual-gating analysis from T0 (black circles; n=14), T1 (grey circles; n=14) and T2 (lightgrey circles; n=14) conditions in (A) CD4+ T cells, (B) CD8+ T cells and (C) CD19+ B cells. The central bar represents the mean ± SEM. One-way Kruskal-Wallis test with Benjamini-Hochberg correction for multiple comparisons was used to test the differences (for CD4+ and CD8+ T cells) among the three groups; q-values are indicated in the figure. For CD19+ B cells was used Mann–Whitney nonparametric test to test the differences among the two groups. Exact p-values are indicated in the figure.
