# Supplementary material for: Effective Treatment of Patients Experiencing Primary, Acute HIV Infection Decreases Exhausted/Activated CD4+ T Cells and CD8+ T Memory Stem Cells
Source: Cells. 2022 Jul 27;11(15):2307. doi: 10.3390/cells11152307 (PMC9367582; doi:10.3390/cells11152307)
Supplement: Supplementary file 1 [file cells-11-02307-s001.zip › Tables S1-S3.pdf]

**Table S1: CD4 T cell subsets identified by the high dimensional data approach**

| | |
|---|---|
| 1. Naïve CD38- | CCR7+ CD45RA+ CD27+ CD28+ CD127+ CD25+ CD95- CD57- PD1- CD38- DR- |
| 2. Naïve CD38+ | CCR7+ CD45RA+ CD27+ CD28+ CD127+ CD25+ CD95- CD57- PD1- CD38+ DR- |
| 3. Tscm CD38+ DR+ PD1+ | CCR7+ CD45RA+ CD27+ CD28+ CD95+ CD127- CD25- CD57- CD38+ DR+ PD1+ |
| 4. CM CD38+ PD1+ | CCR7+ CD45RA- CD27+ CD28+ CD95+ CD127+- CD25+- CD57- PD1+- CD38+ DR+ |
| 5. CM CD38+ PD1- | CCR7+ CD45RA- CD27+ CD28+ CD95+ CD127+- CD25+- CD57- PD1- CD38+ DR- |
| 6. CM CD38- PD1- | CCR7+ CD45RA- CD27+ CD28+ CD95+ CD127+ CD25+- CD57- PD1- CD38- DR- |
| 7. TM CD38- DR+ | CCR7- CD45RA- CD27+ CD28+ CD95+ CD127+- CD25+- CD57- PD1+- CD38- DR+ |
| 8. TM CD38- DR- | CCR7- CD45RA- CD27- CD28+ CD95+ CD127+ CD25+- CD57- PD1- CD38- DR- |
| 9. EM CD38+ DR+ | CCR7- CD45RA- CD27- CD28- CD95+- CD127- CD25+- CD57- PD1+ CD38- DR- |
| 10. EM CD38- DR- CD57+ | CCR7- CD45RA- CD27- CD28- CD95+- CD127- CD25+- CD57- PD1+ CD38- DR- |
| 11. EM CD38+ DR+ CD57+ | CCR7- CD45RA- CD27- CD28- CD95+- CD127- CD25+- CD57+ PD1+ CD38+ DR+ |
| 12. TEMRA | CCR7- CD45RA+ CD27- CD28- CD95+ CD127- CD25+- CD57+ PD1- CD38- DR- |

**Table S2: CD8 T cell subsets identified by the high dimensional data approach**

| | |
|---|---|
| 1. Naïve CD38- | CD45RA+ CD57- CD127+ CD25+- CD95- CD38- DR- CD28+ CCR7+ CD27+ PD1- |
| 2. Naïve CD38+ | CD45RA+ CD57- CD127+ CD25+- CD95- CD38+ DR- CD28+ CCR7+ CD27+ PD1- |
| 3. Tscm CD38+ DR+ PD1+ | CD45RA+ CD57- CD127+ CD25-+ CD95+ CD38+ DR- CD28+ CCR7+ CD27+ PD1+ |
| 4. CM CD38+ DR+ | CD45RA- CD57- CD127+ CD25+- CD95+ CD38+ DR+ CD28+ CCR7+ CD27+ PD1+ |
| 5. TM DR+ | CD45RA- CD57- CD127+ CD25+- CD95+ CD38- DR+ CD28+ CCR7- CD27+ PD1+ |
| 6. TM DR- | CD45RA- CD57- CD127+ CD25+- CD95+ CD38- DR- CD28+ CCR7- CD27+ PD1+ |
| 7. EM CD38+ DR+ CD57- | CD45RA- CD57- CD127- CD25+- CD95+ CD38+ DR+ CD28- CCR7- CD27+ PD1+ |
| 8. EM CD38+ DR+ CD57+ | CD45RA- CD57+ CD127- CD25+- CD95+ CD38+ DR+ CD28- CCR7- CD27+ PD1+ |
| 9. EM CD57+ PD1- | CD45RA- CD57+ CD127- CD25+- CD95+ CD38- DR- CD28- CCR7- CD27- PD1- |
| 10. EM CD57+ PD1+ | CD45RA- CD57+ CD127- CD25+- CD95+ CD38- DR- CD28- CCR7- CD27- PD1+ |
| 11. EM CD57- PD1- | CD45RA- CD57- CD127- CD25+- CD95+ CD38- DR- CD28- CCR7- CD27- PD1- |
| 12. TEMRA CD38- CD57+ | CD45RA+ CD57+ CD127- CD25- CD95+ CD38- DR- CD28- CCR7- CD27- PD1- |
| 13. TEMRA CD38- CD57- | CD45RA+ CD57- CD127- CD25- CD95+ CD38- DR- CD28- CCR7- CD27- PD1- |
| 14. TEMRA CD38+ CD57+ | CD45RA+ CD57+ CD127- CD25- CD95+ CD38+ DR- CD28- CCR7- CD27- PD1- |

**Table S3: B cell subsets identified by the high dimensional data approach**

| | |
|---|---|
| 1. Naïve | IgD+ CD38- IgM+ CD21+ CD27- CD24+- |
| 2. Naïve IgD- | IgD- CD38- IgM+ CD21+ CD27- CD24+- |
| 3. Memory unswitched | IgD+ CD38- IgM+ CD21- CD27+ CD24+ |
| 4. Memory switched | IgD- CD38- IgM- CD21+ CD27- CD24+ |
| 5. Memory IgM ONLY | IgD- CD38- IgM- CD21+ CD27- CD24+ |
| 6. Plasmablasts IgM- | IgD- CD38+ IgM- CD21- CD27+ CD24- |
| 7. Plasmablasts IgM+ | IgD- CD38+ IgM+ CD21- CD27+ CD24- |
| 8. Transitional | IgD+ CD38+ IgM+ CD21+ CD27- CD24+ |
| 9. Exhausted IgD+ IgM+ | IgD+ CD38- IgM+ CD21- CD27- CD24- |
| 10. Exhausted IgD- IgM+ | IgD- CD38- IgM+ CD21- CD27- CD24- |
| 11. Surface- | IgD- CD38- IgM- CD21- CD27- CD24- |
